# Supplementary figures and images for: Reinterpretation of the localization of the ATP binding cassette transporter ABCG1 in insulin-secreting cells and insights regarding its trafficking and function
Source: PLoS One. 2018 Sep 20;13(9):e0198383. doi: 10.1371/journal.pone.0198383 (PMC6147399; doi:10.1371/journal.pone.0198383)

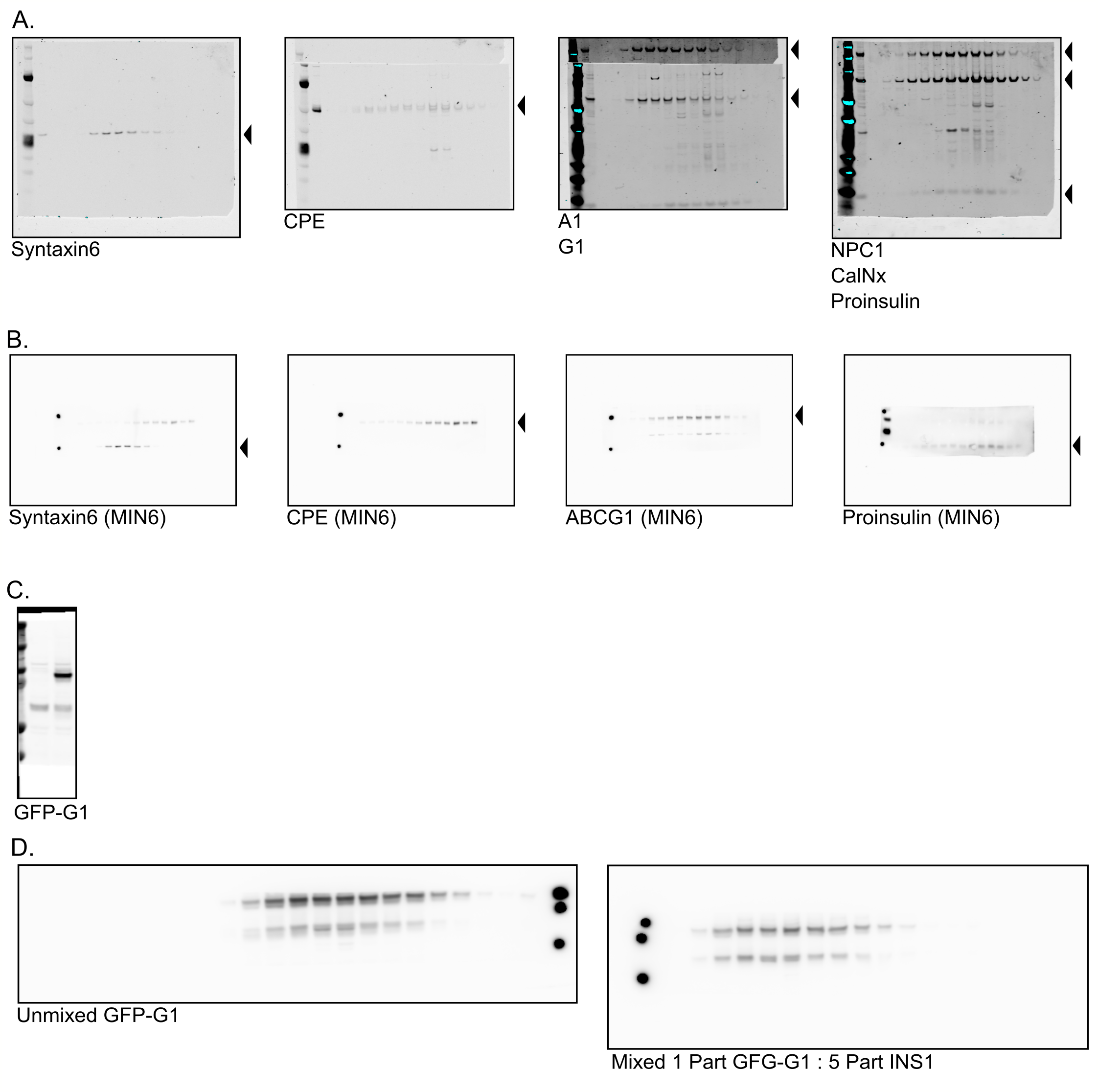

Supplement: S1 Fig — (A) Blots for gradient fractions from INS1 cells presented in Fig 1A. (B) Blots for gradient fractions from MIN6 cells presented in Fig 1B. (C) Blot showing the level of expression of GFP-G1 related to Fig 1C. (D) Blots of gradient fractions for the mixing experiment presented in Fig 1D. (TIF) [file pone.0198383.s001.tif]

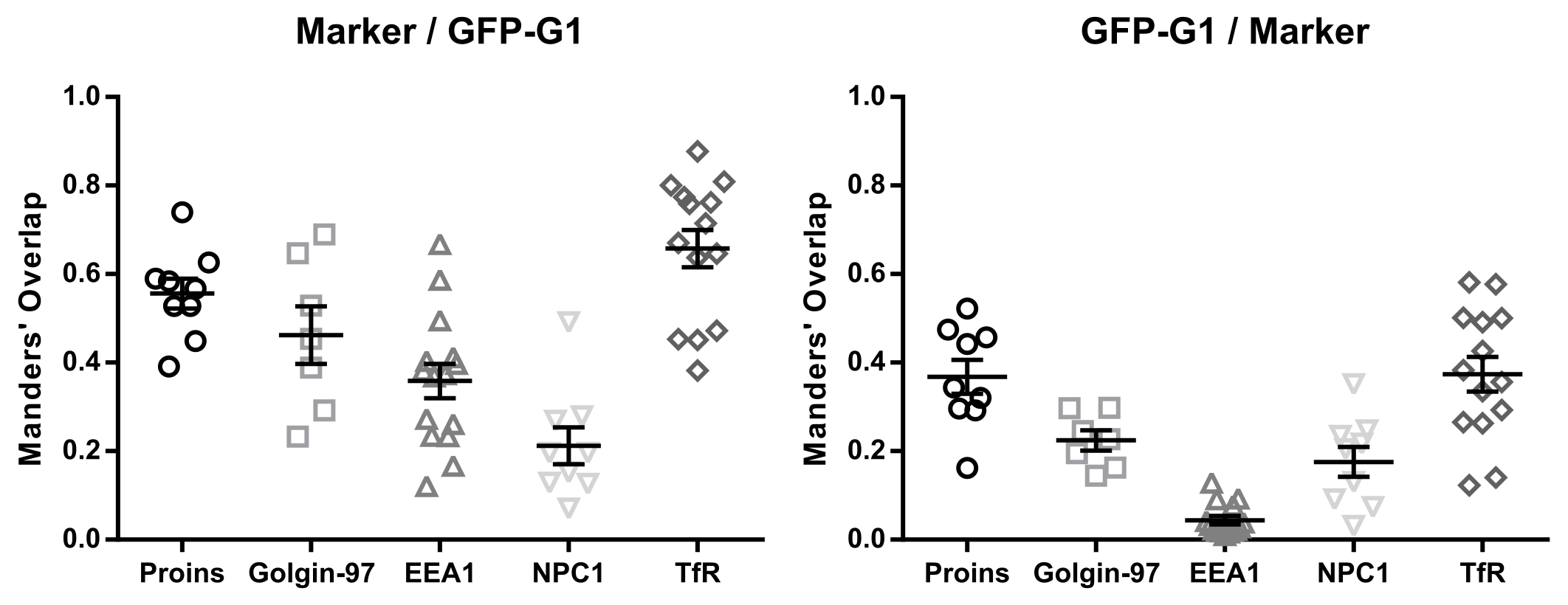

Supplement: S2 Fig — Left panel shows Mander’s overlap measured for Marker/GFP-G1; right panel shows overlap measured for GFP-G1/Marker. Error bars indicate SEM. (TIF) [file pone.0198383.s002.tif]

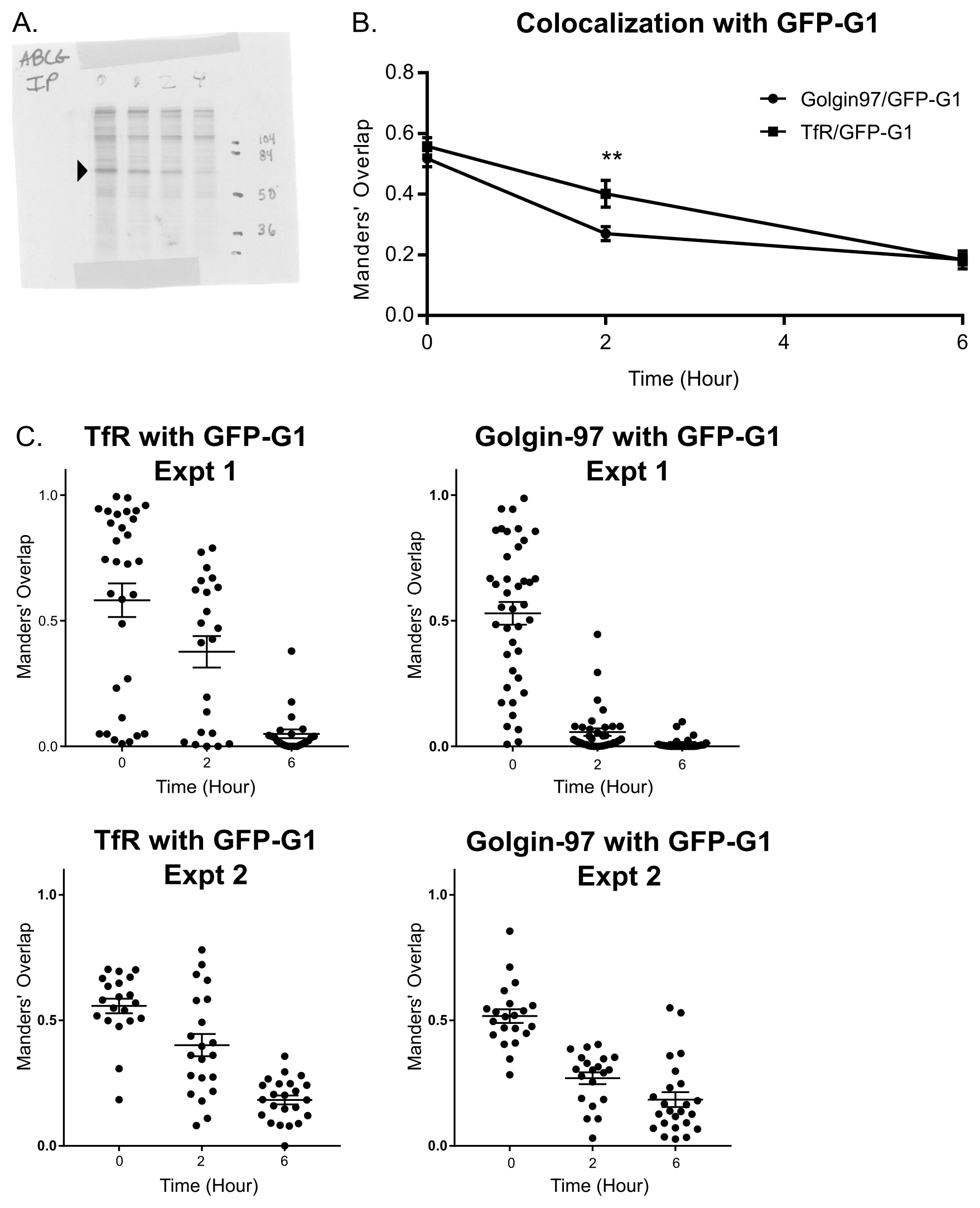

Supplement: S3 Fig — (A) Full x-ray film for immunoadsorption of 35S-labeled ABCG1 shown in Fig 4A. (B) Overlap results of a second experiment identical to that presented in Fig 4E. (C) Full scatter plots for the colocalization data presented in Fig 4E and in Figure B in S3 Fig. (TIF) [file pone.0198383.s003.tif]

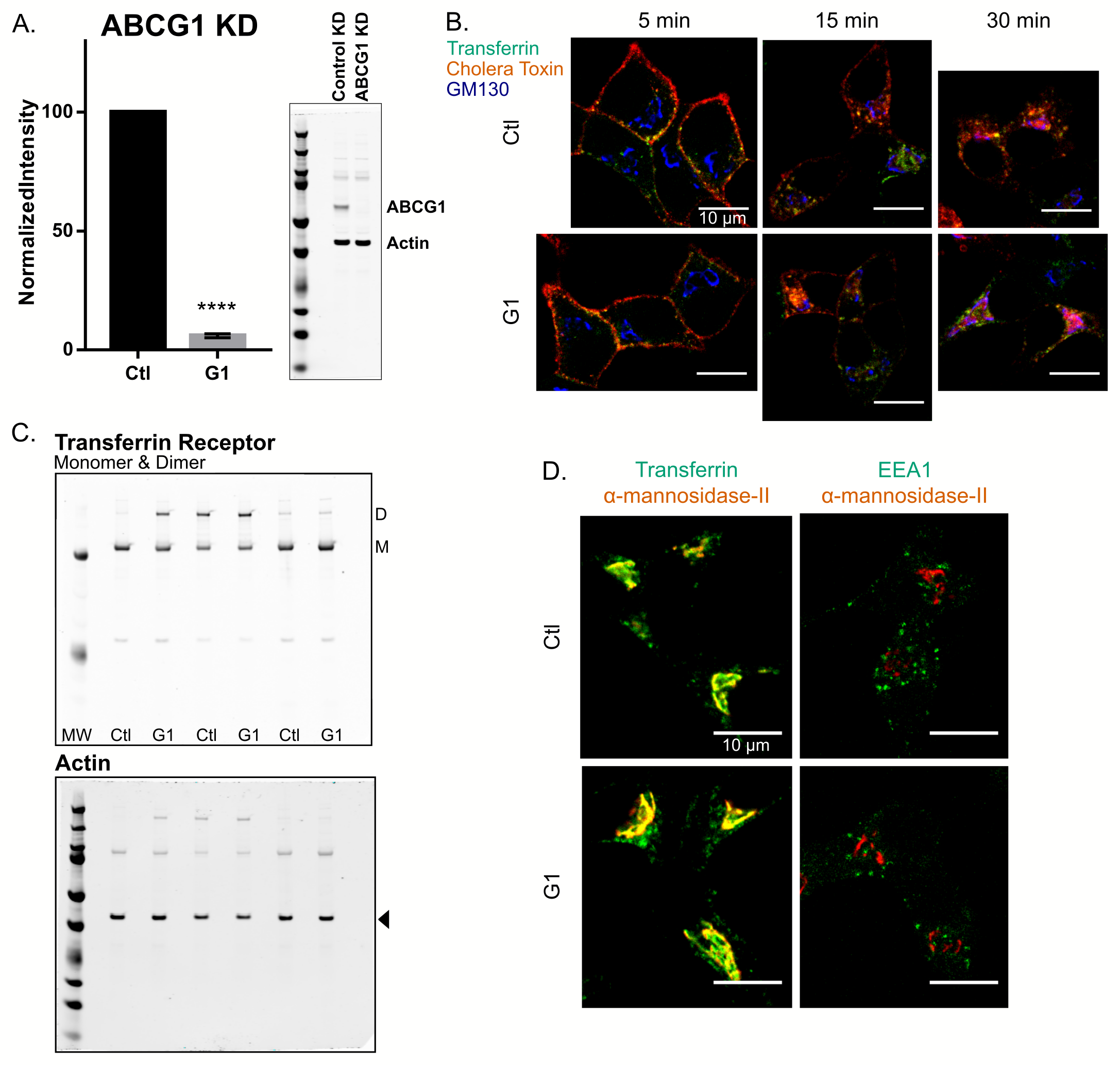

Supplement: S4 Fig — (A) siRNA-mediated knockdown of ABCG1 summarized from four independent experiments accompanied by a sample western blot showing ABCG1 and actin (used for normalization). (B) Example images of Tf-DyLight488 and CTxB-Alexa555 and immunostained GM130 in Control and ABCG1-depleted cells at 5, 15, and 30 min. (C) Full western blots showing results from three (out of four) independent experiments where level of TfR was compared in Control and ABCG1 knockdown samples. (D) Example images of immunostained α-mannosidase-II with either TfR or EEA1. (TIF) [file pone.0198383.s004.tif]

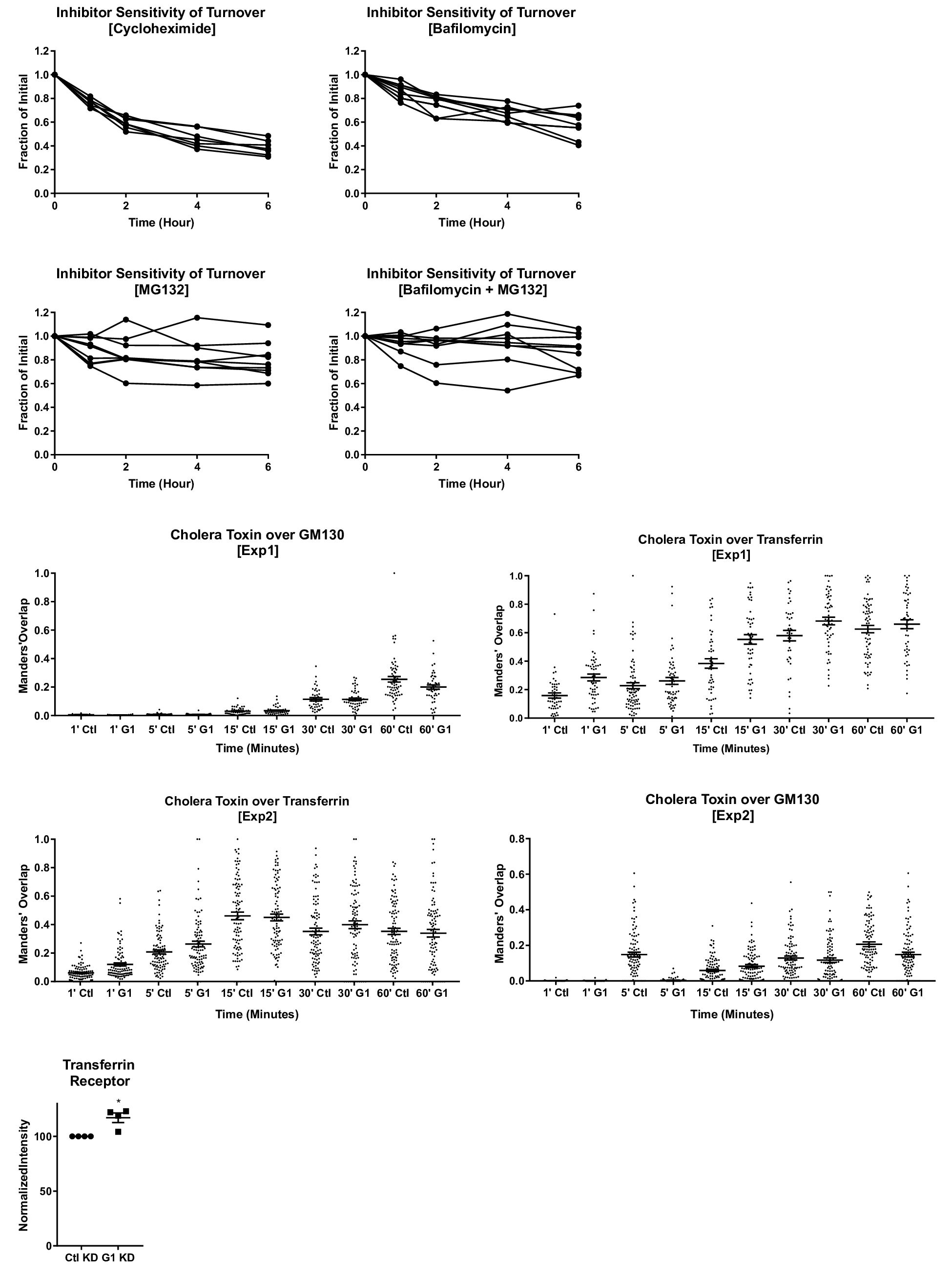

Supplement: S1 File — All data for seven turnover experiments for Fig 4B grouped by treatment; Scatter plots for Fig 5C–5E. (TIF) [file pone.0198383.s007.tif]
